# Comparison of salivary cytokines levels among individuals with Down syndrome, cerebral palsy and normoactive

**DOI:** 10.4317/jced.56336

**Published:** 2020-05-01

**Authors:** Carolina-Hartung Habibe, Rosemeire-Arai Yoshida, Renata Gorjão, Gabriela-Mancia de Gutierrez, Debora Heller, Alexander Birbrair, Maria-Teresa-Botti-Rodrigues Santos

**Affiliations:** 1 DDS, MSc, PhD, Professor, Pediatric Dentistry, Centro Universitário de Volta Redonda, UniFOA, Av. Lucas Evangelista de Oliveira Franco, 866, Volta Redonda. Brazil; 2 DDS, MSc student, Postgraduate Program in Dentistry, Cruzeiro do Sul University, Rua Galvão Bueno, 868 - Liberdade, São Paulo. Brazil; 3 MSc, PhD, Adjunt Professor, Postgraduate Program Interdisciplinary in Health Sciences, Cruzeiro do Sul University, Rua Galvão Bueno, 868 - Liberdade, São Paulo. Brazil; 4 DDS, MSc, Ph.D student, Postgraduate Program in Dentistry, Cruzeiro do Sul University, Rua Galvão Bueno, 868 - Liberdade, São Paulo. Brazil; 5 DDS, MSc, PhD, School of Dentistry, Cruzeiro do Sul University, Rua Galvão Bueno, 868 - Liberdade, São Paulo - SP, 01506-000, Brazil. Experimental Research Center, Albert Einstein Israeli Hospital, São Paulo, São Paulo, Brazil; 6 DDS, MSc, PhD, Department of Radiology, Columbia University Medical Center, New York, New York 622 W 168th St, New York, NY 10032, EUA. Department of Pathology, Federal University of Minas Gerais, Belo Horizonte, Minas Gerais, Brazil; 7 DDS, MSc, PhD, Associate Professor, Individuals with Special Needs, Postgraduate Program in Dentistry, Cruzeiro do Sul University São Paulo, SP, Brazil

## Abstract

**Background:**

Individuals with Down syndrome (DS) present increased susceptibility to infections and high prevalence of periodontal disease. The objective of this study is to evaluate the salivary concentrations of IL-1β, IL-6, IL-8, IL-10, TNFα and IL-12p70 of DS individuals and compare to cerebral palsy (CP) and normoactive patients (all with gingivitis).

**Material and Methods:**

Twenty-two individuals with DS, 24 with CP and 22 normoactive participated in this cross-sectional study. Salivary flow rate, osmolality rate, Oral Hygiene Index, Gingival Index (GI) and salivary inflammatory markers IL-1β, IL-6, IL-8, IL-10, TNFα and IL-12p70 were evaluated. Shapiro-Wilks, Chi-square, ANOVA One-Way and Kruskal Wallis tests were applied with significance level at 5%.

**Results:**

The groups were homogenous for gender, age, and IL12p70 cytokine (*p*>0.05). GI was significantly higher in DS compared to CP and healthy (*p*<0.05). CP presented reduced salivary flow and increased osmolality rate. CP showed significantly higher values for TNFα, IL10, and IL6 compared to DS and normoactive (*p*<0.05). DS and CP presented significantly higher values of IL-1β and IL8 compared to normoactive (*p*<0.05).

**Conclusions:**

Individuals with CP have higher risk to develop periodontal disease due to reduced salivary flow rate, increased salivary osmolality rate and elevated TNFα, IL-10, IL-6 compared to DS.

** Key words:**Cytokines, biomarkers, gingivitis, periodontal diseases, Down syndrome, cerebral palsy, saliva.

## Introduction

Down syndrome (DS) is a frequent genetic chromosomal disorder resulted from the presence of a third chromosome 21 or trisomy 21. Individuals with this condition have physical and functional changes such as intellectual disabilities, heart disease, changes in the immune system that predispose to increased susceptibility to infections (1) and a high prevalence of periodontal disease (2).

Individuals with DS have higher prevalence and severity of periodontal disease than individuals without DS. Several factors contribute to development of periodontal disease in individuals with DS, which is classified as a clinical manifestation of a systemic disease that affect the periodontal supporting tissues (3). The difficulty in performing oral hygiene predisposes the accumulation of biofilm and thus the development of high levels of gingivitis (4). Another fact that could contribute to the presence of periodontal disease in individuals with DS is the impaired host-response (5).

Due to immunological deficiency in DS patients, the infections are more severe, mainly in respiratory system explained by alterations in humoral immunity (5). The abnormalities of the immune system associated with DS include an imbalance in the subpopulations of T and B lymphocytes, with marked decrease of naive T lymphocytes, impaired mitogen-induced T cell proliferation, reduced neutrophil phagocytosis and chemotaxis and reduction in specific antibody responses during immunizations (6) and larger production of inflammatory mediators (7).

Gingivitis is a reversible inflammatory process induced by the presence of microorganisms in the biofilm near the gingival margin (8). The presence of bacterial lipopolysaccharides triggers the inflammatory response of the host, activating polymorphonuclear leukocytes and the secretion of inflammatory mediators such as cytokines and chemokines (9). Proinflammatory cytokines such as interleukin-1 beta (IL-1β), tumor necrosis factor alpha (TNFα) and interleukin-6 (IL-6) are released in response to these inflammatory and infectious stimuli (10).

Saliva has been used as a promising non-invasive diagnostic tool because it is an easily accessible fluid containing proteins, immunoglobulins and formed elements of blood from the gingival tissues (11). The changes in the inflammatory mediators present in saliva reflect the changes that occur in gingival tissue (12). Most studies evaluated the inflammatory cytokines through collection of blood or gingival crevicular fluid in individuals with DS (13). There is a lack in the literature regarding salivary cytokine levels in individuals with DS. This evaluation is important because gingivitis is a disease of high prevalence in individuals with DS and proinflammatory cytokines determination may indicate the immunological status of these individuals.

In this context, the objectives of this study were (i) to evaluate the salivary concentrations of IL-1β, IL-6, IL-8, IL-10, TNFα and the p70 subunit of interleukin-12 (IL-12p70) of DS individuals with gingivitis and compare to individuals with cerebral palsy (CP) and healthy ones (both with gingivitis); (ii) to evaluate and compare salivary flow rate and osmolality values among these individuals. These comparisons were made since individuals with DS and CP present some degree of physical disability that could implicate in oral health issues (14).

The hypothesis of the study was that individuals with DS present higher levels of IL-1β, IL-6, IL-8, IL-10, TNFα and the p70 subunit of interleukin-12 (IL-12p70) cytokines when compared to individuals with CP and normoactive, all with gingivitis.

## Material and Methods

-Ethical statement

This study was reviewed and approved by the Research Ethics Committee of the Centro Universitário de Volta Redonda (CoEPS) Brazil Platform, RJ Brazil (#194,615SP) and by the Research Ethics Committee of the Cruzeiro do Sul University - Brazil Platform, SP Brazil (#1,938,626). Written informed consent was obtained from the legal guardians of each participant after they were informed about the study.

-Study design

A cross-sectional study was conducted with individuals with DS who were referred to Association of Parents and Friends of Exceptional (APAE) and with individuals with CP who received rehabilitation treatment at Assistance Association for the Disabled Children (AACD).

-Subjects

A total of seventy-three individuals with a medical diagnosis of DS (ICD 10 Q90), 58 with a medical diagnosis of CP (ICD 10 G80), and 40 normoactive ones were invited to participate in this study.

Inclusion criteria were individuals with diagnosis of DS, CP and normoactive who presented gingival marginal bleeding (GI) at least 30% of the sites evaluated (15) and age between seven to 18 years old. Progressive or neurodegenerative lesions were excluded, as well as adolescents who were using any medication that interfere with salivary secretion for at least 72 hours prior to salivary collection or had undergone surgical procedures to control the external flow of saliva. Individuals who did not collaborate with the salivary collection or had used an antibiotic in the last month, with symptoms of fever, flu, pains in the body or diarrhea at the moment of collection, or those with some inflammatory condition in the oral mucosa, such as aphthous ulcers were excluded from the study (16).

The following evaluations were performed: inflammatory markers in saliva, salivary flow and osmolality rates, Oral Hygiene Index (17) and Gingival Index (18).

-Saliva collection

Unstimulated saliva samples were collected in the dental assessment sessions as previously described (12). Participants were asked to refrain from eating, drinking liquids or brushing their teeth for at least 1 hour prior to saliva collection. Whole saliva was collected using an absorbent cotton roller (Salivette®; Sarsted, Numbrecht, Germany) positioned on the floor of the mouth for five minutes. The collection was performed with the individuals sitting comfortably in a bright and ventilated room. After collection, the Salivette® was centrifuged at 5,000 rpm for five minutes at 4°C (Hettich Centrifuge, model Universal 320R, Tuttlingen, Germany) and frozen in a freezer at -80°C. After thawing the samples, the salivary flow rate was determined by the ratio volume (mL) produced for 5 minutes.

-Biomarker analysis

The analysis of cytokines in saliva was performed using a CBA Cytokine Inflammatory Kit (Becton Dickinson, CA, USA) for the detection of IL-1β, IL-6, IL-8, IL-10, IL-12p70 and TNFα. All analyses were performed in duplicate.

Briefly, 25 μl of fluorescent particles conjugated to antibodies specific for each cytokine were added to 25 μl of the saliva and incubated for one hour at room temperature away from light. Subsequently, 25 μl of the secondary antibody conjugated to a fluorochrome was added to the mixture and incubated for two hours at room temperature. The results were compared to a standard curve with serially diluted cytokines. The particles were washed to remove the unbound antibodies, resuspended in wash buffer and analyzed using a BD Accuri (BD Biosciences). Data acquisition was performed using BD-Accuri C6 Software, and concentrations were determined using FCAP software v.3.0 (BD Biosciences).

-Saliva osmolality

Saliva osmolality was determined by cooling point depression in an osmometer (Model Vapro Vapor Pressure Osmometer 5600; New Instrument, Washington, DC, USA) as previously described (16).

-Oral Hygiene Index 

Six teeth (four posterior and two anterior) were assessed and scored for each individual according to the Simplified Oral Hygiene Index (OHI-S) (17). For the posterior teeth, the first fully erupted tooth distal to the second premolar or primary molar was examined in each quadrant. For maxillary molars, the buccal surfaces were scored, and for mandibular molars, the lingual surfaces were scored. For anterior teeth, the labial surfaces of the maxillary right and mandibular left central incisors were scored. The OHI-S is a combination of visible plaque and oral calculus indices.

-Gingival index (GI)

The evaluation of the GI (18) was performed using a millimeter plastic periodontal probe (HuFriedy’s Colorvue PerioScreen Kit probe, Chicago, IL, USA), which was gently passed in the gingival margin of all teeth, in reference to the distobuccal papilla, the buccal margin, the mesiobuccal papilla and the lingual / palliative margin. Partially erupted teeth and residual roots were excluded without replacement. The index was calculated by the percentage of the sum of the individual values of each tooth divided by the number of faces examined. Were considered individuals with gingivitis, that ones who presented gingival marginal bleeding at least 30% of the total sites evaluated (15).

-Statistical Analyses

Analyses of descriptive statistics were performed to characterize the sample, calculate measures of central tendency and variability for the quantitative variables. The normality assumption of the quantitative variables was evaluated using the Shapiro-Wilks test. When normal distribution was observed, parametric tests were performed. Otherwise, non-parametric tests were selected to determine the significance of intergroups differences. The Chi-square test was used to analyze the variables of gender, and Kruskal Wallis to analyze cytokines levels.

The ANOVA One-Way test (parametric data) was used to determine significant intergroups differences in relation to the periodontal condition and salivary parameters studied. IBM SPSS Statistics (SPSS for Windows, Version 20.0, Armonk, NY: IBM Corp.) was used for all analyses, with a significance level of 5%.

## Results

The sample power was calculated using the means and standard deviations of TNFα between the groups of individuals with DS (Mean±SD: 1.45±1.92) and individuals with CP (Mean±SD: 7.26±3.22) (OpenEpi online; www.openepi.com). The results showed that at the 95% confidence interval, the G Power was 100%.

The groups were homogenous for gender (*p*=0.457), age (*p*=0.328), and IL12p70 cytokine (*p*=0.322). Although, individuals with DS and CP did not differ regarding OHI-S, the percentage of GI was significantly higher in individuals with DS compared to CP and health ones (*p*-value<0.05) (Table 1).

**Table 1:**
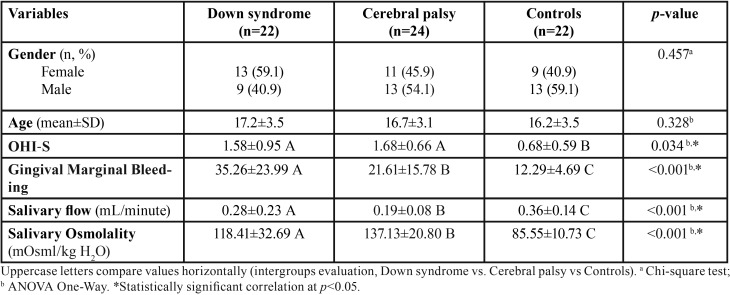
Distribution of individuals with Down syndrome, cerebral palsy and health ones with gingivitis, according to gender, age, oral hygiene, gingival marginal bleeding, salivary flow and osmolality rates evaluated.

Regarding salivary flow and osmolality rates, the CP group presented the worst results with reduced flow and increased osmolality rate. Regarding cytokines the CP group also presented significantly higher values for TNF α, IL10, and IL6 compared to the groups with DS and health ones (*p-value*s<0.05) (Table 1).

In relation to cytokines analysis, DS and CP presented significantly higher values of IL-1β and IL8 compared to the health group (*p*-value<0.05) (Table 2).

**Table 2:**
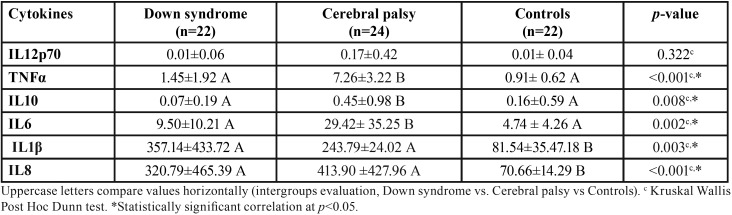
Distribution of individuals with Down syndrome, cerebral palsy and health ones with gingivitis, according to cytokines evaluated.

The DS and CP groups showed a higher percentage of the cytokines IL-1β (DS 77.3%, CP 66.7%), IL-8 (DS: 78.1%, CP: 83.1%) and IL 10 (DS: 56.2%, CP: 64.4%) in relation to the control group. However, for the TNFα and IL6 cytokines, the CP group presented a higher percentage of these cytokines (TNFα: 87.5%; IL6: 83.9%) compared to the control group (Table 2).

## Discussion

To the best of our knowledge, this is the first study to evaluate and compare the salivary concentrations of inflammatory cytokines TNF-α, IL-1β, IL-6, IL-8, IL-10, and IL-12p70 in the saliva of individuals with DS, CP and health ones. Both DS and CP are considered disabilities, and have certain characteristics in common. Individuals with these conditions are likely to face lifelong challenges, including physical challenges, mental and oral health issues (14). Higher IOH-S levels were observed in subjects with DS and CP in relation to healthy individuals. Intellectual and physical disabilities are the main factors that interfere in the performance of oral hygiene, facilitating the accumulation of biofilm and consequently periodontal disease. Thus, it is necessary for these individuals a rigid biofilm control, oral hygiene instructions with toothbrushes with adaptations that facilitate brush handling not only for individuals with impairment but also for their caregiver, and preventive oral health care at regular intervals.

Gingival bleeding showcases important information regarding the presence of periodontal inflammation and has been used as a parameter for clinical evaluation (19). A significant higher value of gingival bleeding was observed in individuals with DS. These results corroborate with findings in the literature related to poor oral hygiene, the compromised immune system and reduced neutrophil chemotaxis in individuals with DS (6).

Among the functions of saliva are the mechanical cleaning and protective functions, essential for the maintenance of oral health (20). The volume of salivary flow and the expression of salivary osmolality contribute to the integrity of dental elements and periodontal health. In this study, it was observed significantly reduced values of salivary flow and high values of salivary osmolality in individuals with CP. These conditions predispose to bacterial accumulation and increase gingival inflammation (21) when compared to DS and health individuals.

Inflammatory cytokines can be evaluated in serum (13) or in saliva (11). In the case of individuals with disability, blood collection to evaluate inflammatory cytokines is generally not an easy task. Furthermore, individuals with CP present sympathetic predominance (22), resulting in vasoconstriction, making venous access more difficult. In this way, saliva has been used since it is ease to collect, contain ions, proteins, immunoglobulins and inflammatory biomarkers (12). Another important point in relation to salivary cytokine analysis is that it may indicate the local secretion of these mediators leading to a better comprehension of the gingival inflammatory status.

The presence of periodontal disease results of the interaction of microbial agents, host defense mechanisms, genetic and environmental factors (23). High levels of IL-1β were found in saliva of individuals with DS and CP in the present study. The secretion of this cytokine is related to cellular alterations such as caspase-1 activation, an important component of inflammation and therefore, leading to activation of inflammatory cells (24). In addition, the neutrophils activation in inflamed gingival tissue may result from the action of IL-1β. The group composed of healthy individuals presented significantly lower values of IL-1β, which could indicate a decrease in neutrophil activation, which characterizes a reduced inflammatory process. These results suggest the involvement of IL-1β in the pathogenesis and progression of periodontal disease, mainly in individuals with DS due to the increased susceptibility to infections, and compromised immune system (25).

Individuals with DS presented similar values for IL-10, TNFα and IL6 when compared to the control group. However, elevated levels of TNF-α, IL10, IL-6, IL-1β and IL-8 were observed in the CP group, which may increase the susceptibility and progression of periodontal disease in these individuals (10). In fact, TNF-α and IL-6 are correlated with the clinical indicators of periodontitis severity as demonstrated by previous studies (26). These cytokines are involved with induction of adhesion molecules that facilitate leukocytes recruitment and augment the inflammatory response, the stimulation of matrix metalloproteinase, and finally bone resorption.

It was observed a significant difference of IL-8 secretion for DS and CP groups in relation to control group. The expression of this chemokine indicates the presence of an inflammatory process, signaling the progression of periodontal disease to apical periodontitis (27,28) for both groups. This chemokine promotes the recruitment of neutrophils (29), the first line of defense for periodontopathogen bacteria and allows the cell to affect phagocytosis and destruction of microorganisms through the production of reactive oxygen species and proteolytic enzymes (30).

These findings provide a possible reason of why individuals with cerebral palsy are at higher risk to develop periodontal diseases. Further research is needed in order to integrate the results with tailored clinical treatment for these patients. The limitations of this study are the use of convenience samples and the absence of inflammatory cytokines determination in serum.

Individuals with cerebral palsy present higher risk to develop periodontal disease due to the reduced salivary flow rate, increased salivary osmolality rate and elevated levels of TNFα, IL-10, IL-6 compared to individuals with individuals with Down syndrome.
